# Supplementary material for: Deciphering Selectivity Mechanism of BRD9 and TAF1(2) toward Inhibitors Based on Multiple Short Molecular Dynamics Simulations and MM-GBSA Calculations
Source: Molecules. 2023 Mar 12;28(6):2583. doi: 10.3390/molecules28062583 (PMC10052767; doi:10.3390/molecules28062583)
Supplement: Supplementary file 1 [file molecules-28-02583-s001.zip › molecules-2236310-supplementary.pdf]

Supporting Information

Deciphering selectivity mechanism of BRD9 and TAF1(2) toward inhibitors based on multiple short molecular dynamics simulations and MM-GBSA calculations

Lifei Wang*, Yan Wang, Yingxia Yu, Dong Liu, Juan Zhao, and Lulu Zhang*

School of Science, Shandong Jiaotong University, Jinan, 250357, China

*Correspondence: wanglf@sdjtu.edu.cn (L.W.); zhanglulu@sdjtu.edu.cn (L.Z).

Table S1. Energetic contributions of individual residues to bindings of three inhibitors to BRD9/TAF1(2).

Complexes[a]		Inhibitors																	
		67B						67C						69G					
	Residues	S_{vdw}	B_{vdw}	S_{ele}	B_{ele}	T_{solv}	T_{tot}	S_{vdw}	B_{vdw}	S_{ele}	B_{ele}	T_{solv}	T_{tot}	S_{vdw}	B_{vdw}	S_{ele}	B_{ele}	T_{solv}	T_{tot}
BRD9	G43	-0.04	-0.23	-0.00	-0.34	0.39	-0.23	-0.09	-0.39	-0.06	-0.17	0.50	-0.21	-0.07	-0.38	-0.03	-0.17	0.34	-0.32
	F44	-2.38	-0.61	-0.42	-1.29	2.65	-2.04	-2.63	-0.80	-0.40	-1.07	2.77	-2.13	-2.86	-0.91	-0.27	-1.21	2.46	-2.79
	F45	-0.66	-0.16	-0.22	-0.08	0.45	-0.67	-0.88	-0.25	-0.12	-0.02	0.53	-0.75	-1.10	-0.25	-0.18	-0.06	0.79	-0.79
	V49	-1.47	-0.15	-0.05	-0.00	-0.04	-1.71	-1.70	-0.36	-0.01	-0.70	0.47	-2.30	-1.50	-0.20	-0.07	-0.12	0.09	-1.80
	T50	-0.05	-0.07	0.00	-0.04	0.17	0.02	-0.16	-0.13	-1.12	-0.34	0.86	-0.89	-0.06	-0.08	0.02	0.04	0.09	0.02
	I53	-1.53	-0.37	0.02	-0.54	0.55	-1.87	-1.57	-0.35	-0.20	-0.35	0.48	-1.98	-1.51	-0.31	-0.06	-0.33	0.34	-1.86
	A54	-0.47	-0.16	-0.00	0.14	-0.07	-0.57	-0.48	-0.19	-0.07	0.16	-0.02	-0.59	-0.42	-0.15	-0.03	0.17	-0.07	-0.51
	A96	-0.38	-0.20	-0.09	-0.00	-0.07	-0.75	-0.53	-0.31	-0.07	0.24	-0.31	-1.00	-0.62	-0.28	-0.07	0.12	-0.26	-1.11
	N100	0.01	0.06	-6.93	0.09	4.06	-2.82	0.22	-0.06	-7.50	0.15	4.13	-3.06	-0.00	-0.06	-6.52	0.08	3.64	-2.86
	Y106	-3.27	-0.16	-0.09	0.03	1.26	-2.22	-2.24	-0.14	0.10	0.12	0.67	-1.49	-3.31	-0.37	-0.25	-0.19	1.84	-2.28
TAF1(2)	W1526	-1.28	-0.28	-0.24	-0.46	1.26	-1.00	-1.00	-0.22	-0.03	-0.30	0.76	-0.79	-0.61	-0.14	-0.21	-0.33	0.67	-0.62
	P1527	-1.47	-0.89	0.19	-1.66	2.08	-1.75	-1.39	-0.88	0.17	-1.33	1.85	-1.57	-1.52	-0.73	-0.45	-0.47	1.47	-1.70
	F1528	-0.85	-0.23	-0.26	0.01	0.43	-0.91	-1.33	-0.30	-0.36	-0.06	0.69	-1.37	-1.25	-0.29	-0.36	-0.12	0.73	-1.29
	V1532	-1.59	-0.50	0.03	-1.15	0.73	-2.47	-1.38	-0.42	0.03	-0.96	0.61	-2.13	-1.09	-0.21	0.01	-0.07	0.03	-1.34
	N1533	-0.49	-0.21	-0.33	-0.96	0.73	-1.26	-0.37	-0.14	-0.33	-0.83	0.73	-0.94	-0.15	-0.14	-0.00	-0.15	0.32	-0.12
	F1536	-1.18	-0.14	-0.31	-0.59	0.96	-1.27	-1.02	-0.18	-0.25	-0.43	0.86	-1.02	-1.50	-0.37	-0.05	-0.18	0.65	-1.45
	V1537	-1.22	-0.12	-0.14	0.14	-0.04	-1.38	-0.95	-0.11	-0.08	0.13	-0.07	-1.08	-1.19	-0.15	-0.03	0.00	0.04	-1.33
	S1579	-0.16	-0.08	0.42	-0.02	-0.28	-0.12	-0.33	-0.18	0.51	0.03	-0.37	-0.33	-0.17	-0.08	0.34	-0.00	-0.20	-0.11
	N1583	0.19	-0.06	-7.51	-0.17	4.42	-3.12	0.14	-0.06	-7.34	-0.15	4.14	-3.27	0.05	-0.06	-6.39	-0.12	3.85	-2.67
	Y1589	-2.44	-0.06	0.18	-0.09	0.89	-1.53	-2.88	-0.09	0.21	-0.15	1.03	-1.88	-3.16	-0.09	0.01	-0.09	1.29	-2.04

[a] All values are expressed in kcal/mol. S_{vdw}: the van der Waals energies of the side chains in residue, B_{vdw}: the van der Waals energies of the backbone atoms, S_{ele}: the electrostatic energies of the side chains in residue, B_{ele}: the electrostatic energies of the backbone atoms, T_{solv}: the solvation energies of residues, T_{tot}: the binding energies of residues.

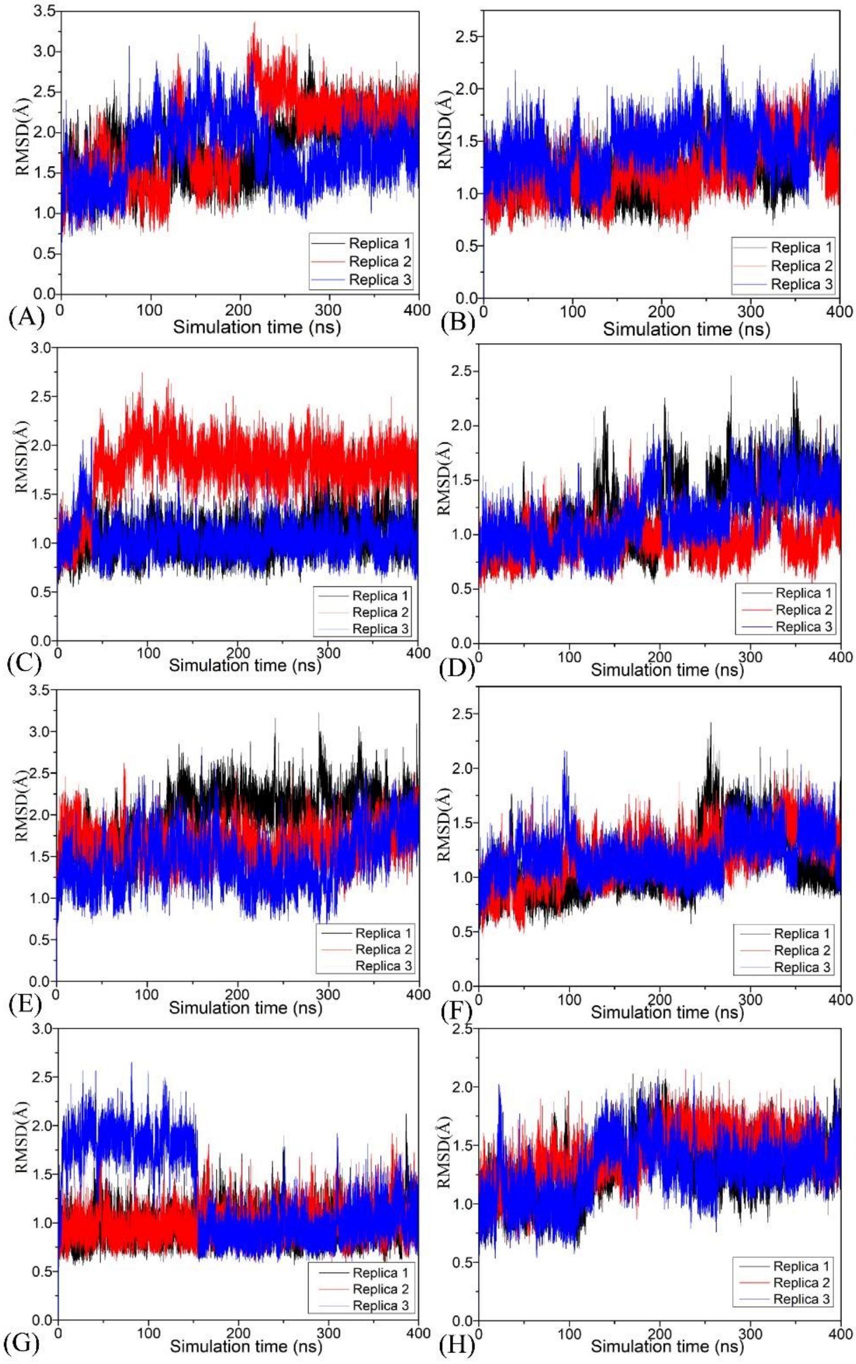

Figure S1. Root-mean-square deviations (RMSDs) of backbone atoms in BRD9 and TAF1(2) computed by using MSMD trajectories of three replicas: (A) the *apo* BRD9, (B) the *apo* TAF1(2), (C) the 67B-BRD9 complex, (D) the 67B-TAF1(2) complex, (E) the 67C-BRD9 complex, (F) the 67C-TAF1(2) complex, (G) the 69G-BRD9 complex, and (H) the 69G-TAF1(2) complex.

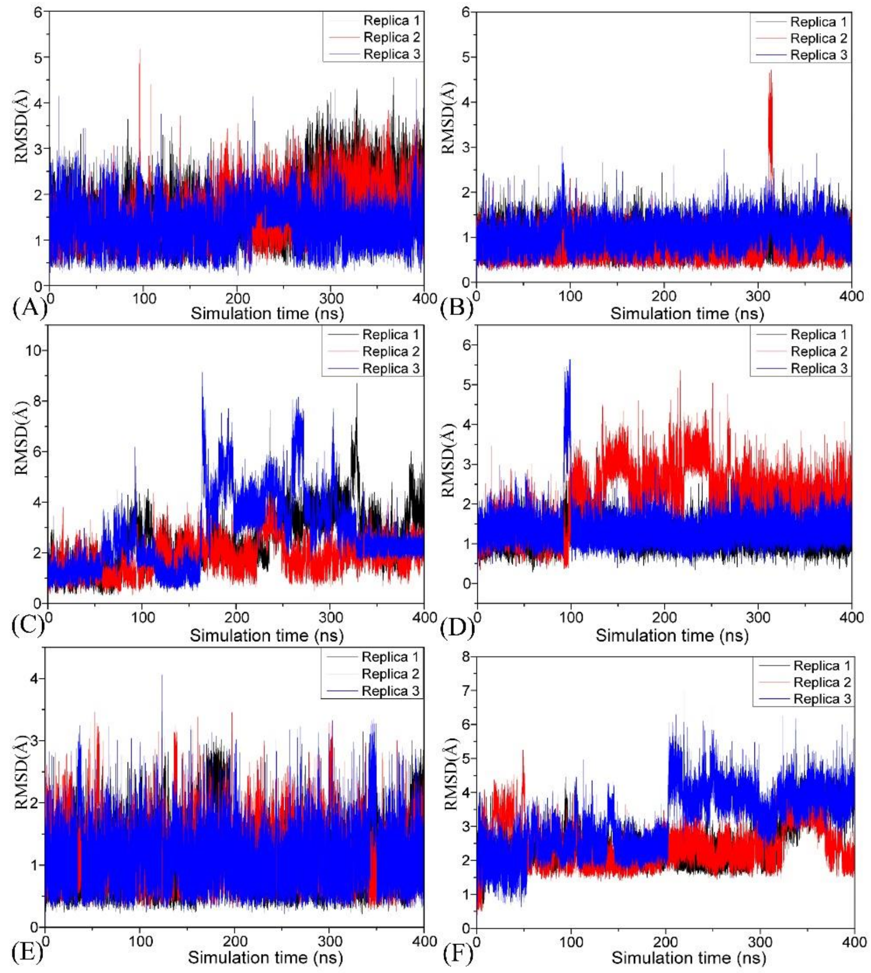

Figure S2. Root-mean-square deviations (RMSDs) of ligands complexed with BRD9 and TAF1(2) computed by using MSMD trajectories of three replicas: (A) the 67B-BRD9 complex, (B) the 67B-TAF1(2) complex, (C) the 67C-BRD9 complex, (D) the 67C-TAF1(2) complex, (E) the 69G-BRD9 complex and (F) the 69G-TAF1(2) complex.

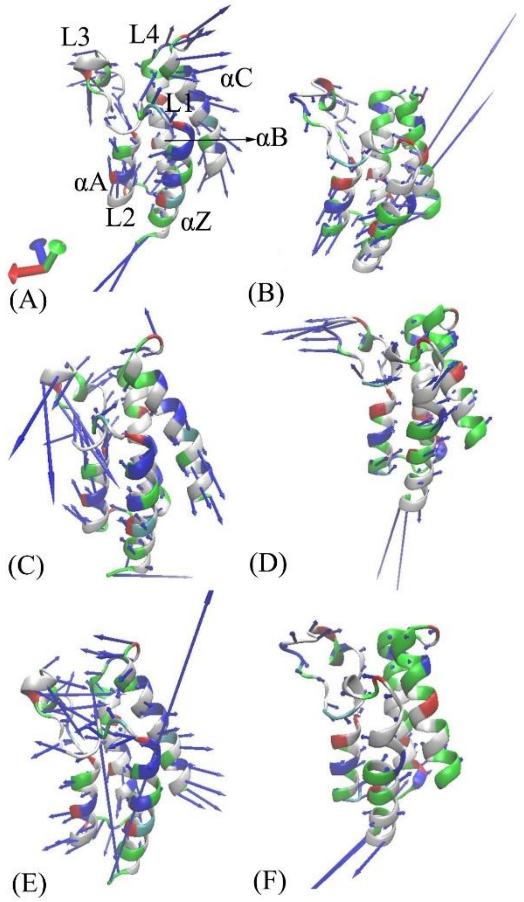

Figure S3. Concerted movements of the structural domain revealed by the first component PC1 obtained from the principal component analysis based on the single joined trajectories: (A) the 67B–BRD9 complex, (B) the 67B–TAF1(2) complex, (C) the 67C–BRD9 complex, (D) the 67C–TAF1(2) complex, (E) the 69G–BRD9 complex, and (F) the 69G–TAF1(2) complex.

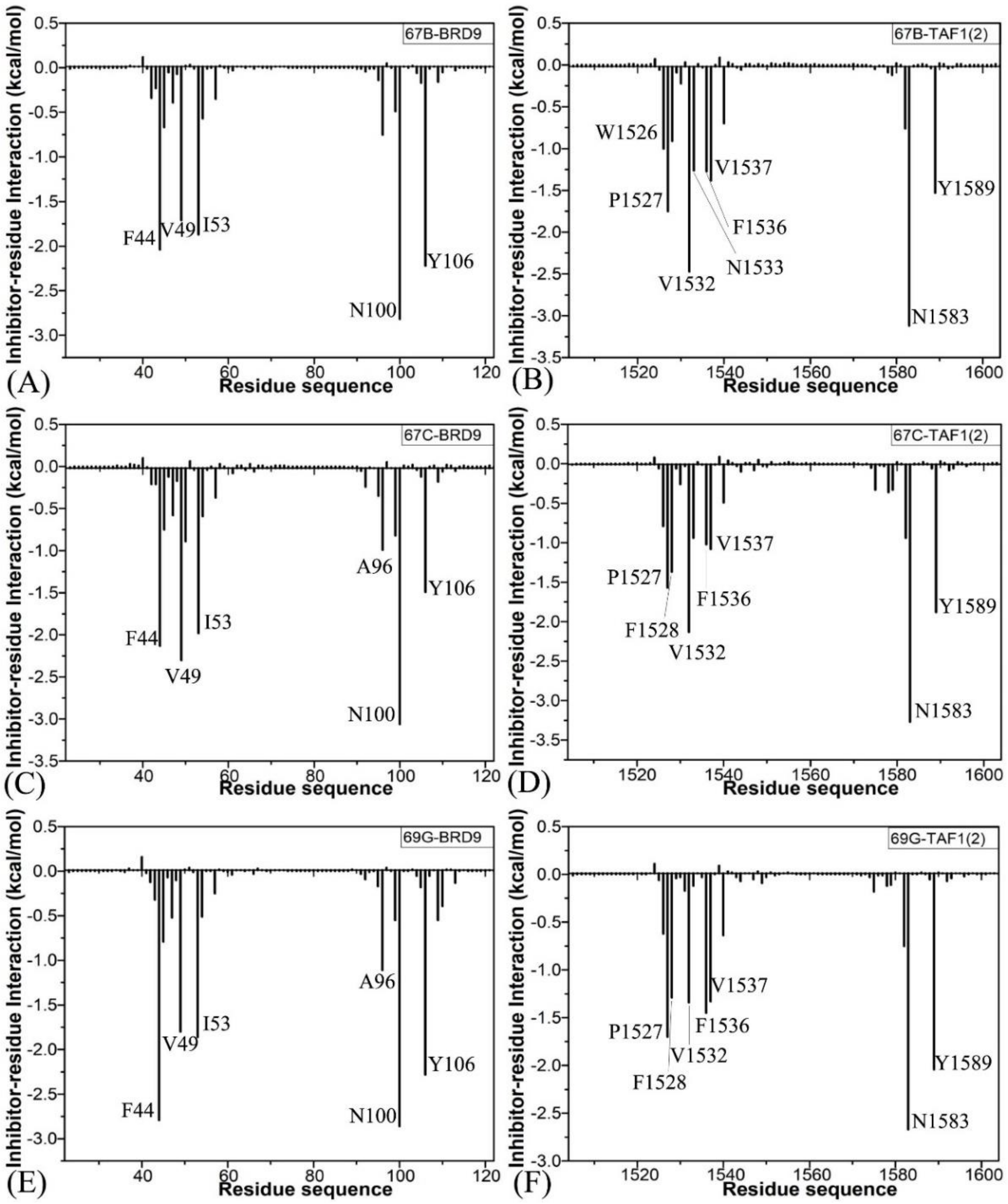

Figure S4. Inhibitor–residue interactions calculated by using the residue-based free energy decomposition approach—only residues stronger than 1.0 kcal/mol were marked: (A) the 67B–BRD9 complex, (B) the 67B–TAF1(2) complex, (C) the 67C–BRD9 complex, (D) the 67C–TAF1(2) complex, (E) the 69G–BRD9 complex, and (F) the 69G–TAF1(2) complex.

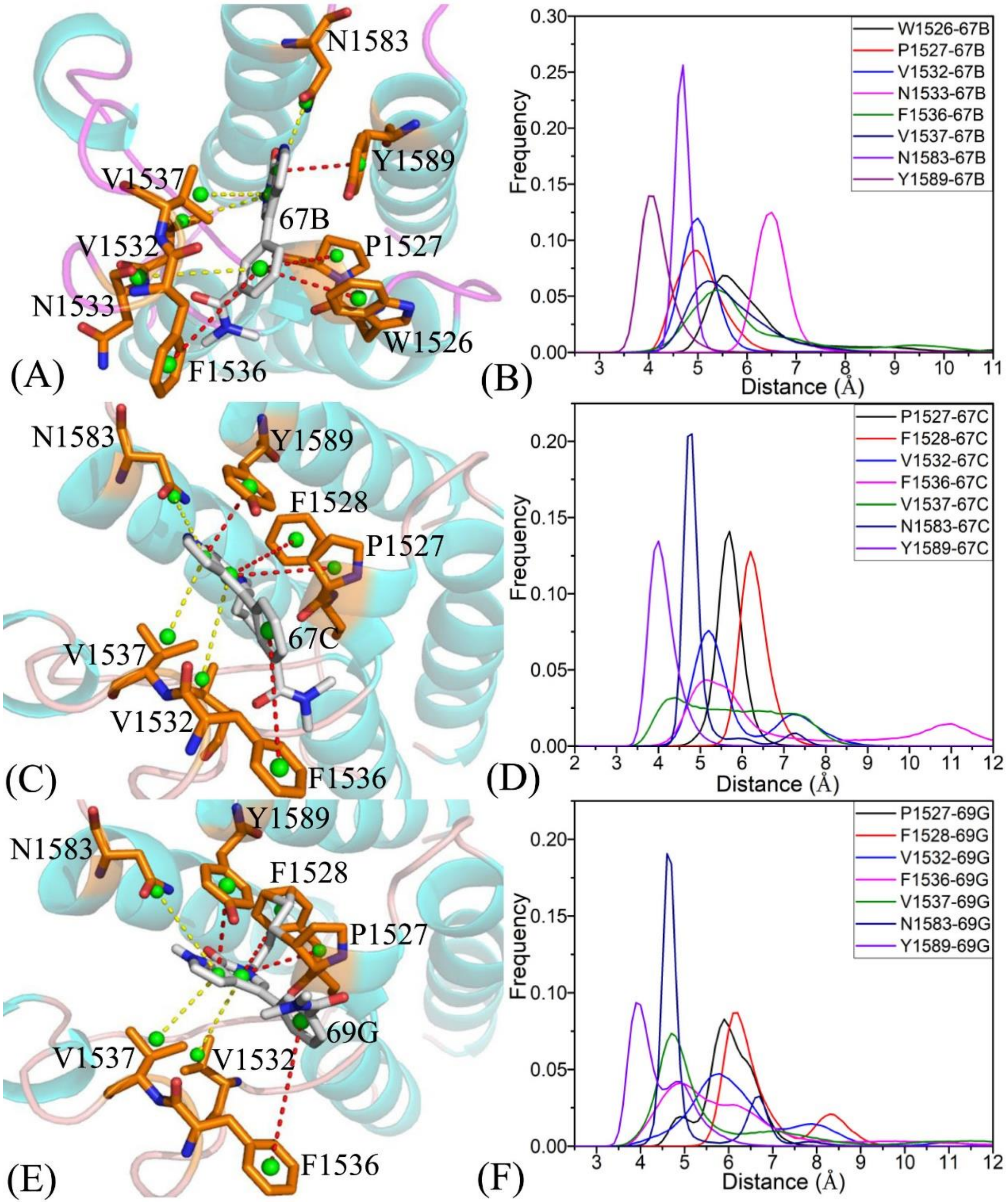

Figure S5. Hydrophobic interactions and frequency distribution of the distance involved in the interactions of inhibitors with important residues: (A) the 67B–TAF1(2) complex; (B) RDF of 67B–TAF1(2); (C) the 67C–TAF1(2) complex; (D) RDF of 67C–TAF1(2); (E) the 69G–TAF1(2) complex; (F) RDF of 69G–TAF1(2). The frequencies of the distances between atoms involving significant interactions were calculated by using the integrated MSMD trajectories of the last 900 ns. The yellow dashed lines indicate the CH–π interactions and the red dashed lines indicate the π–π interactions.

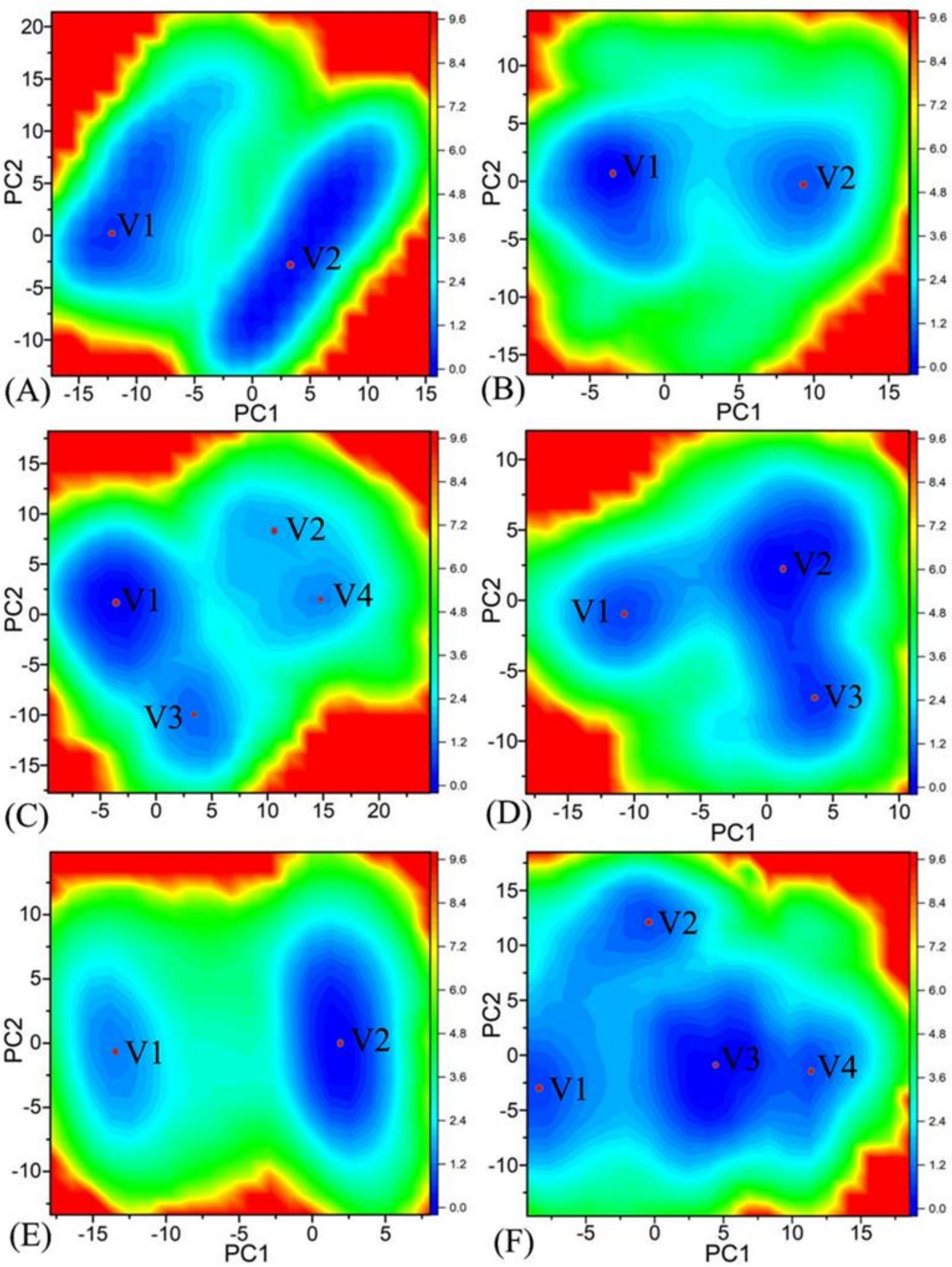

Figure S6. Free energy landscapes constructed by using projections of the single joined MSMD trajectories corresponding to the first two principal components, PC1 and PC2, from the diagonalization of covariance matrix: (A) the 67B–BRD9 complex, (B) the 67B–TAF1(2) complex, (C) the 67C–BRD9 complex, (D) the 67C–TAF1(2) complex, (E) the 69G–BRD9 complex, and (F) the 69G–TAF1(2) complex.
